# Exploring the analgesic effect of artificial dura mater as a carrier for local hydromorphone delivery in posterior lumbar interbody fusion: a randomized controlled trial

**DOI:** 10.3389/fphar.2026.1816434

**Published:** 2026-05-25

**Authors:** Jian Miao, Ruiming Deng, Tingyu He, Ziqiang Dong, Xianwei Jin, Weibo Zhong

**Affiliations:** 1 Department of Anesthesiology, Ganzhou Maternal and Child Healthcare Hospital, Ganzhou, Jiangxi, China; 2 Department of Anesthesiology, Ganzhou People’s Hospital, Ganzhou, Jiangxi, China; 3 Department of Anesthesiology, The First Affiliated Hospital of Nanchang University, Nanchang, Jiangxi, China

**Keywords:** artificial dura mater, gelatin sponge, hydromorphone, local delivery, posterior lumbar interbody fusion

## Abstract

**Objective:**

Carrier-based local hydromorphone delivery using absorbable materials represents an innovative strategy; however, comparative studies on various carriers remain scarce. This study aims to evaluate and compare the clinical efficacy of artificial dura mater versus gelatin sponge as carriers for hydromorphone in patients undergoing posterior lumbar interbody fusion (PLIF).

**Methods:**

This single-center, prospective, randomized controlled trial was conducted at Ganzhou People’s Hospital, with planned enrollment of patients scheduled for single-level PLIF surgery. Participants were randomly assigned to either the gelatin sponge group (Group G) or the artificial dura mater group (Group A). At the conclusion of the surgery, the carrier material was soaked with 0.4 mg of hydromorphone hydrochloride and subsequently placed evenly onto the dural surface. The primary outcome was overall analgesic efficacy during movement within 72 h postoperatively. Secondary outcomes included the overall analgesic efficacy at rest within 72 h postoperatively, Visual Analog Scale (VAS) scores at rest and during movement at 24, 48, and 72 h postoperatively, patient satisfaction, hemodynamic parameters, adverse reactions, analgesic consumption, postoperative Quality of Recovery-15 (QoR-15) score, and Pittsburgh Sleep Quality Index (PSQI) score.

**Results:**

Between February 2025 and July 2025, Seventy-four patients were randomized, and 71 were included in the modified intention-to-treat (mITT) analysis (Group G: n = 35; Group A: n = 36). Group A showed a modest reduction in the area under the curve (AUC) of the time-VAS curve during movement from PACU to 72 h (Median difference, −24; 95%CI, −36 to 0; *P* = 0.045) and lower movement-related VAS at 72 h (*P* = 0.004), with favorable exploratory PSQI (*P* = 0.044)/QoR-15 scores (*P* = 0.022). Finally, no significant between-group difference was observed in adverse events, analgesic satisfaction, or postoperative analgesic consumption (*P* > 0.05).

**Conclusion:**

Artificial dura mater-based local hydromorphone delivery may alleviate movement-evoked pain at 72 h after PLIF.

## 1 Introduction

Lumbar degenerative disease represents a significant global health challenge, leading to chronic pain and functional impairment in affected individuals. The prevalence of PLIF procedures has surged, driven by an aging population and resulting in increased healthcare expenditures and socioeconomic burdens (Deshpande et al., 2025). However, PLIF surgery is characterized by its high invasiveness, necessitating extensive muscle dissection, manipulation of bony structures, and handling of nerve roots, which often results in severe acute postoperative pain (Lee et al., 2024). This intense pain not only contributes to patient suffering but also serves as a major barrier to early postoperative recovery (Fiasconaro et al., 2020). Epidemiological studies reveal that patients with chronic musculoskeletal pain face a 1.91-fold higher risk of developing cardiovascular diseases compared to those without such pain (Oliveira et al., 2020). Additionally, pain-induced reductions in mobility may elevate the risk of deep vein thrombosis, pulmonary infections, and muscle atrophy. Furthermore, emerging evidence indicates a strong association between acute pain and cognitive impairment, including delirium (Ma et al., 2023), particularly in elderly patients, with potential underlying mechanisms involving neuroinflammation and central sensitization. Therefore, ensuring effective and safe analgesia following PLIF surgery is essential for facilitating early recovery in patients.

Currently, postoperative analgesia for spinal surgery primarily relies on multimodal strategies, often combining systemic opioids (Trenchfield et al., 2024), nonsteroidal anti-inflammatory drugs (NSAIDs) (Chaibhuddanugul et al., 2024), and regional block techniques (Zhang et al., 2023). Although patient-controlled epidural analgesia (PCEA) demonstrates reliable efficacy (Schenk et al., 2006), the use of indwelling catheters may pose risks such as infection, epidural hematoma, catheter displacement or occlusion, hypotension, and motor blockade. The high management and monitoring requirements associated with PCEA limit its effective use in certain clinical scenarios or patient populations. While intravenous patient-controlled analgesia (IV-PCA) offers convenience, postoperative nausea and vomiting remain significant concerns (Wu et al., 2011). In recent years, the application of local infiltration analgesia and regional nerve blocks has increased (Li et al., 2019); however, the duration of analgesia is often limited to within 24 h, making it challenging to adequately cover the peak pain period of 72 h postoperatively.

In this context, our team investigated carrier-based local delivery of hydromorphone at the surgical site for postoperative pain management in spinal surgery (Jin et al., 2024). The core concept involves the use of bioabsorbable materials placed intraoperatively at the surgical site as drug “depots,” which enable continuous and slow local release of medication to achieve long-lasting targeted analgesia while minimizing systemic exposure and side effects. Similarly, the innovative study by Yang et al. (Yang et al., 2020) demonstrated that infiltrating a mixture of ropivacaine, dexamethasone, and vitamin B12 into absorbable gelatin sponges placed epidurally after lumbar surgery could significantly prolong analgesic duration. However, the use of gelatin sponges as a carrier may present certain limitations. Their loose structure and high water absorption capacity may lead to rapid drug elution or diffusion in surgical sites with abundant blood and tissue exudate, resulting in unstable drug release. After the initial burst release, subsequent maintenance may be insufficient. In the study by Yang et al., gelatin sponges prepared through different cross-linking methods exhibited high porosity and excellent water absorption, but their material strength and degradation performance varied significantly among samples (Yang et al., 2018). This indicates that, although gelatin sponges offer advantages in water absorption and biocompatibility, their inadequate mechanical strength may affect stable drug release. Additionally, their low mechanical strength and tendency to fragment may compromise the uniformity of drug coverage.

Artificial dura mater is a well-established product in neurosurgery and spinal surgery for the repair or reinforcement of the dura mater, offering excellent biocompatibility, biodegradability, and certain mechanical properties. Compared to gelatin sponge, its microstructure is denser and more organized, which may confer superior drug-loading capacity and controlled release characteristics (Sun et al., 2024). Its degradation cycle is significantly longer than that of gelatin sponge, theoretically enabling better alignment with the acute postoperative pain period and potentially accommodating longer analgesic cycles, thus providing a smoother and more sustained drug release profile (Ramot et al., 2020; Goncalves et al., 2015). Therefore, artificial dura mater may emerge as a novel local drug delivery carrier with enhanced performance. In terms of drug selection, our preliminary studies have confirmed the efficacy of hydromorphone. As a semi-synthetic potent μ-opioid receptor agonist, its equivalent ratio to morphine is approximately 1:6 (Rodrigues et al., 2021). Relevant studies (Li et al., 2024) indicate that at equianalgesic doses, hydromorphone and morphine exhibit similar overall side effect profiles, although some research suggests that hydromorphone may have a lower tendency to induce respiratory depression (Clemens and Klaschik, 2008; Chang et al., 2006), demonstrating favorable safety in acute pain management. However, despite the promising concept of localized sustained-release analgesia, there is currently a lack of high-quality randomized controlled trials comparing different biomaterials as carriers for opioid drugs. Studies that systematically evaluate multiple dimensions, particularly regarding analgesic effects, systemic inflammation regulation, neurocognitive protection, and comprehensive rehabilitation, are even rarer.

Based on the aforementioned background, we propose the core scientific hypothesis that in PLIF surgery, the use of artificial dura mater as a carrier for local hydromorphone delivery may provide superior and more prolonged epidural analgesia compared with traditional gelatin sponges.

## 2 Methods

### 2.1 Study design

This study is a single-center, prospective, randomized controlled clinical trial that received approval from the Medical Ethics Committee of Ganzhou People’s Hospital (Ethics Approval No.: TY-ZKY2024-094-01). Prior to the enrollment of the first patient, the trial was registered with the Chinese Clinical Trial Registry (Registration No.: ChiCTR2500096777). Before the commencement of the trial, the study protocol was thoroughly explained to the patients and their families, including the relevant treatment measures, and written informed consent was obtained from all participants. We strictly adhered to the Declaration of Helsinki and complied with the Consolidated Standards of Reporting Trials (CONSORT) reporting guidelines.

Eligible patients scheduled to undergo single level posterior lumbar fusion under general anesthesia at Ganzhou People’s Hospital from February 2025 to July 2025 were selected for this study. The inclusion criteria were as follows: patients scheduled for elective posterior lumbar fusion with fenestration decompression, aged between 18 and 74 years; the surgical area involved single segments, and patients had an ASA classification of I-III. The exclusion criteria included: patients who refused to participate in the study; those with a history of alcoholism; individuals with visual or hearing impairments that hinder normal communication; patients with mental health disorders or those using psychotropic medications; individuals with abnormal liver or kidney function; a history of previous spinal surgery or spinal tumor surgery; allergies to non-steroidal drugs, opioids, or any materials used in the study (such as gelatin or collagen); long-term use of analgesic medications; or individuals deemed unsuitable for participation by the investigator. Regarding exclusion and dropout criteria, patients will be excluded if they undergo a change in surgical approach or any unplanned concomitant procedure during the index surgery, and they will receive no further study intervention. During the study implementation, if patients fail to cooperate or voluntarily withdraw, they will be classified as dropouts.

#### 2.1.1 Randomization and blinding

Participants were randomly assigned in a 1:1 ratio to either the gelatin sponge group (Group G) or the artificial dura mater group (Group A) using computer-generated randomization tables with blinding (as shown in Figure 2). To conceal the allocation, the study numbers and group assignments were placed in sealed, opaque envelopes by operating room nurses who were not involved in the study.

Prior to the commencement of the trial, a designated nurse who was not involved in any other study procedures sequentially opened the sealed, opaque envelopes according to the order of participant enrollment to obtain the allocation. Subsequently, a designated anesthesiologist (not involved in perioperative management, outcome assessment, or follow-up) prepared the corresponding carrier-based local hydromorphone delivery systems based on that allocation for delivery to the operating surgeon. Because the two carrier materials were visually distinguishable, the intervention-preparing anesthesiologist and the operating surgeon were aware of group allocation; however, they were not involved in postoperative outcome assessment or statistical analysis. The anesthesia team (anesthesiologists involved in perioperative management and follow-up), patients, outcome assessors, and statisticians were all blinded to group allocation to ensure data objectivity. An independent data and safety monitoring committee oversaw the trial procedures to guarantee that group allocation remained concealed throughout the analysis phase. The group allocation remained undisclosed until the conclusion of the trial.

### 2.2 Material preparation

This study utilized various pharmaceutical agents, including Hydromorphone Hydrochloride Injection (Yichang Humanwell Pharmaceutical Co., Ltd., 2 mL:2 mg), Propofol Emulsion Injection (Jiangsu Yingkang Bio-Pharmaceutical Co., Ltd., 20 mL:200 mg), Sufentanil Citrate Injection (Yichang Humanwell Pharmaceutical Co., Ltd., 1 mL:50 μg), and Remifentanil for Injection (Yichang Humanwell Pharmaceutical Co., Ltd., 1 mg). Additionally, Rocuronium Bromide (Emeishan Tonghui Pharmaceutical Co., Ltd., 5 mL:50 mg), Sevoflurane (Shanghai Hengrui Pharmaceutical Co., Ltd.), Penehyclidine hydrochloride (Chongqing Pharscin Pharmaceutical Co., Ltd., 1 mL:1 mg), Etomidate (Jiangsu Ehwa Pharmaceutical Co., Ltd., 10 mL:20 mg) and Cisatracurium Besylate (Shanghai Hengrui Pharmaceutical Co., Ltd., 5 mL:10 mg) were included in the analysis. The study also employed Sodium Chloride Injection (10 mL:0.09 g), 500 mL 0.9% Sodium Chloride Injection, and 2.5mL/5mL/10 mL Disposable Sterile Syringes. Furthermore, Artificial Dura Mater (Beijing Bonse Technology Co., Ltd., 30*40 mm), Absorbable Gelatin Sponge (Jinling Pharmaceutical Co., Ltd., 60*20 mm), and an Electronic Infusion Pump were utilized. The research incorporated Noradrenaline (1 mL:2 mg), Atropine Sulfate Injection (1 mL:0.5 mg), Flurbiprofen Axetil Injection (Grand Medical Nutraceutical Science Co., Ltd., 5 mL:50 mg), and Sterile Gloves.

### 2.3 Anesthesia and monitoring

Both groups of patients undergoing elective lumbar fusion surgery routinely followed preoperative fasting for 8 h and fluid restriction for 2 h. After entering the operating room, patients underwent routine monitoring of blood pressure (BP), heart rate (HR), electrocardiogram (ECG), and SpO2. The circulating nurse established peripheral intravenous access in the upper limb using a 20-gauge indwelling needle and set up radial artery blood pressure monitoring before anesthesia induction. Both groups received anesthesia induction with Penehyclidine hydrochloride 0.5mg, Sufentanil 0.2 μg/kg, Propofol Injection 1–2 mg/kg, Etomidate 0.2 mg/kg and Rocuronium Bromide 0.6 mg/kg. Anesthesia maintenance was achieved with Sevoflurane, target-controlled infusion (Beijing SiLuGaO) of Propofol 1.0-3.0 μg/ml, and Remifentanil 2–5 ng/mL, with intermittent intravenous administration of Cisatracurium for muscle relaxation. Sevoflurane was discontinued 30 min before the end of surgery, Propofol was stopped 5–10 min prior, and Remifentanil was ceased at the conclusion of surgery, with an additional intravenous dose of Sufentanil 0.1-0.2 μg/kg administered to prevent postoperative breakthrough pain. Continuous intraoperative monitoring of the Bispectral Index (BIS) was performed, with BIS values maintained between 40 and 60. Maintenance medication was adjusted based on BIS values and vital signs to keep mean blood pressure and heart rate fluctuations within 20% of baseline values. If hemodynamic parameters exceeded 20% of baseline values and the Surgical Pleth Index (SPI) was above 50, an additional 5–10 μg of sufentanil was administered to maintain appropriate anesthetic depth during surgery. Ventilation parameters were set as follows: tidal volume (VT) 6–8 mL/kg, inspired oxygen concentration (FiO2) 60%, respiratory rate (f) 10-20 breaths/min, and inspiratory-to-expiratory ratio 1:2. Respiratory parameters were adjusted to maintain intraoperative PaCO2 between 35-45 mmHg. Intraoperative fluid replacement was administered according to the 4-2-1 rule to meet preoperative and intraoperative physiological requirements, with adjustments made based on the patient’s circulatory changes during surgery. The anesthesia management during the perioperative period was carried out by an experienced anesthesiologist who was blinded to the group allocation.

### 2.4 Research intervention (carrier placement)

**FIGURE 1:**
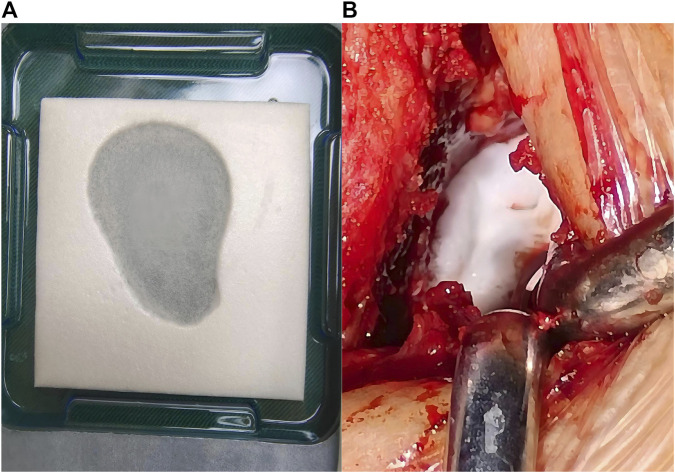
**(A)** Hydromorphone was dripped onto the artificial dura mater. **(B)** Make the artificial dura mater fully cover the spinal membrane.

To prepare the hydromorphone hydrochloride injection, first dilute it with 0.9% sodium chloride injection to a total volume of 10 mL. Next, using a 2.5 mL syringe equipped with a No. 3 needle, draw 2 mL (equivalent to 0.4 mg) of the diluted hydromorphone hydrochloride solution. In the gelatin sponge group (Group G), evenly drip 2 mL of hydromorphone hydrochloride onto a gelatin sponge (6 cm × 2 cm × 0.5 cm) using the syringe. Ensure that the medication is fully absorbed by the gelatin sponge. In the artificial dura mater group (Group A), similarly drip 2 mL of hydromorphone hydrochloride onto a dura mater patch (3 cm × 4 cm) with the syringe. Ensure that the medication is fully absorbed by the patch (as illustrated in Figure 1A). All preparation of the drug and saturation of the gelatin sponge or dura mater patch were performed by a designated anesthesiologist who was not involved in perioperative management, outcome assessment, or follow-up, to ensure consistency. Each patient’s dural sac integrity was carefully examined. In Group G, the surgeon uniformly covers the dura mater with the gelatin sponge soaked in hydromorphone hydrochloride, ensuring complete coverage upon placement. In Group A, the surgeon smoothly applies the dural patch over the dura mater (as depicted in Figure 1B). After carrier placement, both groups received a layer of dry gelatin sponge over the hydromorphone-infiltrated carrier (either artificial dura mater or gelatin sponge) and within the lateral recess. Drainage tubes were then positioned in the corresponding surgical segment. Finally, the paraspinal muscles were sutured, and complete wound closure was achieved within 30 min.

### 2.5 Postoperative analgesia regimen

Upon completion of the surgery, an intravenous analgesic pump was connected to the patient. All pumps utilized the same formulation of sufentanil (2 μg/kg) diluted with 0.9% normal saline to a total volume of 100 mL, with a background infusion rate of 0.5 mL/h, a patient-controlled analgesia (PCA) dose of 2 mL, and a lockout interval of 15 min. Patients were permitted to self-administer additional boluses as needed for effective pain management. Following the surgical procedure, patients were transferred to the Post Anesthesia Care Unit (PACU) for recovery from anesthesia and monitoring of vital signs. The endotracheal tube was removed once patients regained consciousness and met the extubation criteria. Continuous monitoring was maintained until the modified Aldrete score reached ≥9, at which point patients were transferred back to the general ward. The first postoperative pain VAS score was assessed concurrently. If the patient’s postoperative VAS score was four or higher, they were allowed to self-administer a single dose of patient-controlled analgesia via the intravenous analgesic pump. Should the pain persist after multiple attempts and the VAS score remained at four or higher, a 50 mg intravenous injection of flurbiprofen axetil was administered as rescue analgesic therapy.

### 2.6 Data collection and outcomes

The patient’s subjective sleep quality was evaluated using the Pittsburgh Sleep Quality Index (PSQI) 1 day before surgery, the baseline physiological and psychological recovery status was assessed using the Quality of Recovery-15 (QoR-15) scale, and the preoperative pain score was evaluated using the VAS. Postoperative VAS scores, heart rate (HR), and mean arterial pressure (MAP) were assessed at the following time points: at PACU discharge (T0), and at 24 h (T1), 48 h (T2), and 72 h (T3) postoperatively. All data were objectively recorded by anesthesiologists who were blinded to the group allocation.

#### 2.6.1 General information

The general characteristics of patients in both groups were analyzed, including age, gender, height, weight, SPO2 levels, preoperative PSQI score, QoR-15 score, platelet count, hemoglobin concentration, white blood cell count, and C-reactive protein (CRP) levels. Additionally, surgical conditions were assessed, encompassing operation time, anesthesia duration, blood loss, fluid infusion volume, and urine output.

#### 2.6.2 Primary outcome

The overall analgesic effect within 72 h postoperatively was compared between the two groups during movement, which is the time-weighted area under the VAS score curve during movement. The area under the curve (AUC) for the time–VAS curve is calculated using the trapezoidal method. The calculation formula is as follows: AUC = ∑(VAS_a_ + VAS_b_) × (t_b_ - t_a_) ÷ 2, where VASa and VASb represent the VAS scores corresponding to two adjacent postoperative observation time points t_a_ and t_b_ (t_b_ > t_a_) respectively.

The baseline VAS_0_ _h_ refers to the VAS score obtained in the PACU. The scale ranges from 0 to 10, with higher values denoting greater pain intensity. VAS scores during movement are collected by blinded follow-up personnel at the designated intervals [PACU (0 h), 24 h, 48 h, and 72 h]. The primary outcome measures are the cumulative AUC from PACU to 72 h (AUC_PACU-72h_) during movement.

$$\text{Cumulative }{\text{AUC}}_{\text{PACU}-72\;h}=\left[\left({\text{VAS}}_{0\;h}+{\text{VAS}}_{24\;h}\right)\times 24÷2\right]+\left[\left({\text{VAS}}_{24\;h}+{\text{VAS}}_{48\;h}\right)\times 24÷2\right]+\left[{\text{VAS}}_{48\;h}+{\text{VAS}}_{72\;h})\times 24÷2\right]$$

Assessment of movement-related pain: The patient was instructed to grasp the evaluator’s hand or the bed rail and turn to the opposite side. Assistance was provided with equipment and a pillow as needed. The patient was then asked to cough three times and use the incentive spirometer for deep breathing. Upon completion, movement-related pain was assessed using the VAS (Strutz et al., 2019; Booker et al., 2022).

#### 2.6.3 Secondary outcomes

The area under the cumulative AUC at rest during the period from PACU to 72 h (AUC_PACU-72 h_); VAS scores of patients recorded at PACU discharge, and at postoperative time points T1, T2, and T3; Ramsay sedation scores documented during the analgesia period at T1; MAP and HR measured at T0, T1, T2, and T3; Adverse reactions experienced by patients within 72 h post-surgery, including postoperative nausea and vomiting (PONV), somnolence, respiratory depression, and skin itching; Total frequency of Patient-Controlled Analgesia (PCA), total sufentanil dosage, and rescue doses of flurbiprofen axetil administered within 72 h post-surgery; The evaluation of PSQI score at T3 (the PSQI was used only to assess sleep during the first 3 days after surgery); Documentation of postoperative awakening time, use of antagonists, the time when patients began to move independently, the time of first ambulation, total length of postoperative hospital stay, and timing of first gas passage and bowel movement; Assessment of QoR-15 scores for patients at T1 and T3; A satisfaction survey regarding analgesia, conducted using a five-point Likert scale at T3.

### 2.7 Sample size estimation

Preliminary experimental results indicated that the AUC of VAS scores during movement, measured from the PACU to 72 h (AUC_PACU-72 h_), was 286.8 (SD 34.62) in the control group and 260.4 (SD 31.01) in the experimental group. With a two-sided significance level of α = 0.05 and 90% power (1−β = 0.90), assuming a 1:1 allocation ratio, the minimum required total sample size calculated using PASS 15 software was 66 participants (33 per group). To account for potential attrition due to changes in clinical conditions, a 10% dropout rate was factored in. A total of 74 patients were initially recruited for the study. No interim analyses were planned or conducted.

### 2.8 Statistical analysis

Data processing was conducted in accordance with a pre-specified data management and statistical analysis plan. Statistical analyses were performed using the Statistical Package for the Social Sciences version 26.0 (SPSS). The Kolmogorov–Smirnov test was employed to assess the normality of variable distributions. Normally distributed continuous data are presented as mean (standard deviation, SD) and were compared using independent-samples t-test or repeated-measures analysis of variance (RMANOVA), as appropriate. Non-normally distributed data are reported as median (Q1, Q3) and were analyzed using the Mann–Whitney U test. Categorical variables are presented as counts (percentages, %) and were analyzed using Fisher’s exact test. All primary analyses were performed in the modified intention-to-treat (mITT) population, defined as all patients who underwent randomization to single-level lumbar fusion under general anesthesia and did not withdraw consent for the use of their data. Patients were excluded from the mITT analysis if they underwent a change in surgical approach or any unplanned concomitant procedure during the index surgery. To assess the robustness of the findings, sensitivity analyses were performed on baseline data and primary outcomes using the intention-to-treat (ITT) population (all randomized patients). The Hodges–Lehmann method was used to estimate median differences with corresponding 95% confidence interval (CI). Between-group differences are expressed as mean difference (MD) for parametric analyses and median difference for non-parametric analyses (Group A minus Group G). All statistical tests were two-sided, and a *P*-value <0.05 was considered statistically significant.

The primary outcome was reported as median (Q1, Q3) and analyzed using the Mann–Whitney U test. Primary outcome data were missing for three excluded patients who did not receive the assigned intervention. These missing values were handled using multiple imputation by chained equations (MICE) to assess their impact on the primary outcome (AUC_PACU-72 h_). The imputation model included: primary outcome, treatment group, baseline VAS, age, sex, height, weight, BMI, preoperative PSQI score, and preoperative QoR-15 score. Due to the inability of SPSS to automatically pool results from non-parametric tests (Mann-Whitney U) across multiply imputed datasets, between-group comparisons of the primary outcome (AUC_PACU-72 h_) in the sensitivity analysis were conducted using a linear regression model with group allocation as the independent variable. A total of 20 imputed datasets were generated, and pooled estimates of mean difference and 95% confidence intervals were obtained from linear regression using Rubin’s rules. For secondary outcomes, continuous data conforming to a normal distribution are expressed as mean (standard deviation, SD) and compared using repeated-measures analysis of variance (ANOVA). Non-normally distributed data are reported as median (Q1, Q3) and analyzed using the Mann–Whitney U test. Categorical variables are expressed as counts (%), analyzed using Fisher’s exact test, and the relevant risk ratio is calculated.

## 3 Results

**FIGURE 2:**
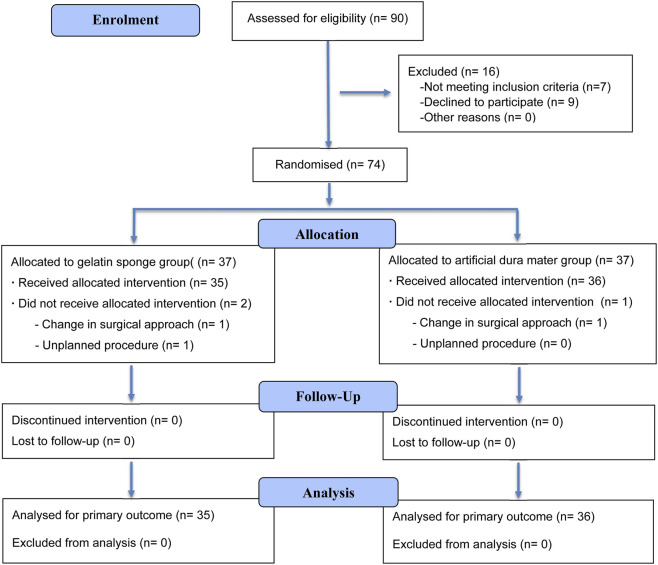
Study Flow Diagram. Group G, gelatin sponge group; group A, artificial dura mater group.

Among the 90 eligible patients selected, 16 (9 declined to participate, two took analgesics, two had a history of spinal surgery, one could not communicate normally, one was allergic to non-steroidal drugs and one had abnormal liver function) were excluded according to the exclusion criteria. Among the 74 patients who underwent randomization, three were excluded from the mITT analysis: two because of changes in surgical approach and one because of an unplanned concomitant procedure performed for severe bleeding during the index surgery. We thus had 71 patients in our mITT population (as shown in Figure 2).

### 3.1 Comparison of baseline characteristics between the two groups of patients

Seventy-four patients were randomized, and 71 were included in the mITT analysis: gelatin sponge group (group G, n = 35; mean (SD) age, 57.86 (8.57) years) and artificial dura mater group (group A, n = 36; mean (SD) age, 55.56 (9.12) years). There was no significant difference between group G and group A in age, gender, height, weight, oxygen saturation, quality of recovery score, preoperative PSQI score, QoR-15 score, platelet count, hemoglobin and other general conditions (*P* > 0.05) (Table 1). The baseline characteristics of the mITT population are shown in Table 1. The full randomized set showed comparable baseline characteristics (Supplementary Table S1).

**TABLE 1:** Demographic and perioperative characteristics of the mITT population.

Characteristic	Patients, mean (SD)		*Z/T/χ2*	*P*
	Group G (n = 35)	Group A (n = 36)		
Age, y	57.86 (8.57) [40–71]	55.56 (9.12) [34–74]	1.095	0.277
Height, median (Q1, Q3), cm	160 (151–167)	162 (155–168)	−0.502	0.616
Weight, median (Q1, Q3), kg	60 (55–66)	60 (55–68)	−0.161	0.872
WBC, 10^9/L	6.71 (1.90)	7.27 (2.56)	−1.042	0.301
HGB, g/L	130.63 (14.93)	135.67 (15.18)	−1.410	0.163
PLT, 10^9/L	235.86 (74.10)	255.67 (75.37)	−1.116	0.268
Albumin, g/L	40.23 (2.94)	41.33 (3.60)	−1.408	0.164
CRP, median (Q1, Q3), mg/L	1.73 (1.40–3.28)	1.81 (1.36–3.82)	0.952	0.344
BMI, kg/m^2^	23.82 (3.02)	23.46 (2.51)	0.543	0.589
Infusion volume, median (Q1, Q3), ml	2000 (1500–2000)	1800 (1500–2000)	−0.128	0.898
Urine volume, median (Q1, Q3), ml	350 (300–500)	300 (200–400)	−1.696	0.090
Blood loss, median (Q1, Q3), ml	100 (100–200)	100 (100–200)	−0.733	0.464
SPO_2_, median (Q1, Q3), %	98 (97–99)	98 (98–99)	−0.292	0.771
Surgical duration, median (Q1, Q3), min	163 (135–189)	150 (139–184)	−0.644	0.519
Preoperative PSQI score, median (Q1, Q3)	8 (6–9)	7.5 (6–9)	−0.589	0.556
Preoperative QoR-15 score, median (Q1, Q3)	145 (143–147)	144 (143–147)	−0.759	0.448
Preoperative RestVAS score, median (Q1, Q3)	1 (1–2)	1 (1–2)	−0.030	0.976
Diabetes, no. (%)	2 (5.7)	2 (5.6)	<0.01	>0.99
Hypertension, no. (%)	6 (17.1)	11 (30.6)	1.753	0.185
Gender				
Male, no. (%)	16 (45.7)	19 (52.8)	0.354	0.552
Female, no. (%)	19 (54.3)	17 (47.2)		

Group G, gelatin sponge group; group A, artificial dura mater group; mITT, modified intention-to-treat; PLT, platelet; HGB, hemoglobin; WBC, white blood cells; SPO2, pulse oxygen saturation; BMI, body mass index; CRP, C-reactive protein; SD, standard deviation; RestVAS, visual analog scale at rest; PSQI, pittsburgh sleep quality index; QoR-15, Quality of Recovery-15; Compared with group G.

### 3.2 Primary outcome: the time-weighted area under the VAS score curve during movement (AUC_PACU-72 h_)

**FIGURE 3:**
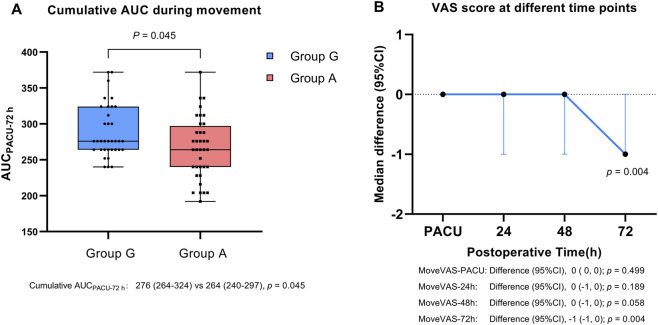
Group G, gelatin sponge group; group A, artificial dura mater group; VAS, Visual Analogue Scale; AUC, Area under the curve; PACU, Post-anaesthesia care unit. Comparisons of the overall postoperative analgesic efficacy as indicated by the cumulative AUC of the VAS scores and postoperative time. **(A)** Intergroup comparison of the AUC_PACU-72 h_ during movement. Box plots display median, Q1, Q3, and full range (minimum to maximum), with all individual data points overlaid. **(B)** Median difference and 95% confidence interval (Hodges-Lehmann estimate) of VAS scores at each time point. The dashed horizontal line at Y = 0 indicates no difference, and error bars represent 95% confidence intervals. The connecting line between time points is for visual guidance only.

Mann–Whitney U test was used to analyze AUC_PACU-72_ _h_, and the results showed that compared with group G, the AUC_PACU-72 h_ during movement in group A was reduced (Median difference, −24; 95% CI, −36 to 0; *P* = 0.045). The results were robust in a prespecified sensitivity analysis that included all patients who underwent randomization (ITT sensitivity analysis with multiple imputation: AUC_PACU-72 h_ during movement: MD, −20.93 [95% CI, −39.81 to −2.05], *P* = 0.030) (Table 2; Figure 3).

**TABLE 2:** Comparison of pain related indicators between the two groups.

Outcomes	Patients, median (Q1, Q3)		Difference/RR (95%CI)	*Z/t/χ2*	*P*
	Group G (n = 35)	Group A (n = 36)			
Cumulative AUC during movement					
AUC_PACU-72 h_ (mITT)	276 (264–324)	264 (240–297)*	−24 (−36, 0)	−2.001	0.045
Sensitivity analysis (ITT, MI), mean	288.96	267.98*	−20.93 (−39.81, −2.05)	−2.172	0.030
Cumulative AUC at rest					
AUC_PACU-72 h_ (mITT)	204 (192–228)	192 (156–225)	−24 (−36, 0)	−1.496	0.135
Sensitivity analysis (ITT, MI), mean	204.69	187.43	−17.21 (−38.80, 4.39)	−1.562	0.118
Comparison of VAS scores among all patients at different time points (mITT)					
RestVAS-PACU	2 (2–2)	2 (1–2)	0 (0, 0)	−1.132	0.258
RestVAS-24 h	3 (3–3)	3 (2–3)	0 (0, 0)	−1.076	0.282
RestVAS-48 h	3 (3–3)	3 (2–3)	0 (−1, 0)	−1.645	0.100
RestVAS-72 h	3 (2–3)	2 (2–3)	0 (−1, 0)	−1.774	0.076
MoveVAS-PACU	3 (3–4)	3 (3–4)	0 (0, 0)	−0.677	0.499
MoveVAS-24 h	4 (4–5)	4 (4–4.75)	0 (−1, 0)	−1.314	0.189
MoveVAS-48 h	4 (4–5)	4 (3–4)	0 (−1, 0)	−1.897	0.058
MoveVAS-72 h	4 (3–4)	3 (3–4)*	−1 (−1, 0)	−2.868	0.004
Comparison of PCA frequency and flurbiprofen axetil usage in all patients (mITT)					
PCA frequency within 72 h	11 (9–14)	9 (8–12)	−1 (−2, 0)	−1.224	0.221
Dosage of flurbiprofen axetil	0 (0–0)	0 (0–0)	0 (0, 0)	−1.026	0.305
Use of flurbiprofen axetil, no. (%)	8 (22.9)	5 (13.9)	0.608 (0.220, 1.678)	0.954	0.329
Total dosage of sufentanil, ug	71.68 (64.80–75.40)	70.16 (58.50–81.46)	−2.08 (−8.76, 6.12)	−0.558	0.577
Overall patient satisfaction with pain relief (mITT)					
Patient satisfaction	5 (4–5)	5 (4–5)	0 (0, 0)	−0.916	0.360
Number and proportion of patients with different satisfaction scores (mITT)					
5, no. (%)	18 (51.4)	22 (61.1)	1.188 (0.785, 1.798)	0.676	0.411
4–5, no. (%)	29 (82.9)	32 (88.9)	1.073 (0.887, 1.297)	0.534	0.465

Mean difference (95% CI) from linear regression based on 20 multiply imputed datasets (pooled estimates using Rubin’s rules). For all AUC, and VAS outcomes, median differences and 95% CI, were estimated using the Hodges-Lehmann method. In the presence of tied observations, the confidence interval may extend to or include zero while the associated *P*-value from the Mann-Whitney U test remains below or approaches 0.05. This is a recognized limitation of the estimator and does not represent a calculation error. Group G, gelatin sponge group; group A, artificial dura mater group; Median difference = Group A− Group G. Negative values favor Group A. RR, Group A/Group G. RR > 1 favors Group A. RR, relative risk; CI, confidence interval; AUC, area under the curve; mITT, modified intention-to-treat; ITT, intention-to-treat; MI, multiple imputation; PACU, Post-anaesthesia care unit; PCA, patient controlled analgesia; RestVAS, visual analog scale at rest; MoveVAS, visual analog scale during movement. Compared with group G.

* *P* < 0.05.

### 3.3 Secondary outcomes

#### 3.3.1 The time-weighted area under the VAS score curve at rest (AUC_PACU-72 h_)

Mann–Whitney U test was used to analyze AUC_PACU-72_ _h_, and the results showed that there was no significant difference in AUC_PACU-72 h_ at rest (Median difference, −24; 95% CI, −36 to 0; *P* = 0.135). Sensitivity analyses using the ITT population yielded consistent results (Table 2).

#### 3.3.2 Details of VAS at each time point and pain related outcomes

Compared with group G, there was no significant difference in the VAS score of group A at rest; During movement, there was a difference only at 72 h (Median difference, −1; 95% CI, −1 to 0; *P* = 0.004). However, there were no significant differences in the total number of PCA frequency within 72 h, the total dosage of sufentanil, the use rate of flurbiprofen axetil and patient satisfaction with pain relief (*P* > 0.05) (Table 2).

#### 3.3.3 Adverse reactions and other efficacy indicators

Fisher’s exact test was used to analyze the incidence of adverse reactions. The results showed that there was no significant difference in the incidence of adverse reactions such as pruritus, drowsiness, respiratory depression and PONV between group G and group A (*P* > 0.05). Furthermore, there were no statistically significant differences between the two groups in postoperative eating time, time to ambulation, or length of hospital stay. However, the PSQI score of group G was higher than that of group A (Median difference, −1; 95% CI, −1 to 0; *P* = 0.044) (Table 3). Finally, by comparing the mean arterial pressure (MAP) and heart rate (HR) of the two groups at different time points, Repeated-measures analysis of variance (RMANOVA) revealed that there was no significant difference in hemodynamic indexes between the two groups (*P* > 0.05) (Table 4).

**TABLE 3:** Comparison of adverse reactions and postoperative recovery between the two groups.

Outcomes	Patients, no. (%)		Difference/RR (95% CI)	*χ2*	*P*
	Group G (n = 35)	Group A (n = 36)			
Pruritus	9 (25.7)	10 (27.8)	1.080 (0.500, 2.336)	0.039	0.844
Drowsiness	4 (11.4)	3 (8.3)	0.729 (0.176, 3.025)	0.002	0.969
Respiratory depression	0 (0)	0 (0)	—	—	—
PONV	5 (14.3)	3 (8.3)	0.583 (0.151, 2.258)	0.174	0.676
PSQI score at T3, median (Q1, Q3)	6 (5–7)	6 (5–6.75)*	−1 (−1, 0)	−2.012	0.044
Ramsay score	2 (2–2)	2 (2–2)	0 (0, 0)	−0.702	0.482
Exhaust time, median (Q1, Q3), h	25 (20–40)	28 (20.25–39.5)	1 (−3, 5)	−0.553	0.580
Eating time, median (Q1, Q3), h	9 (6–10)	7.5 (6–10)	0 (−1, 1)	−0.513	0.608
The time of activities, median (Q1, Q3), d	3 (2–3)	3 (2–3)	0 (0, 0)	−1.607	0.108
Postoperative hospital stay, median (Q1, Q3), d	8 (7–10)	8 (6–9.75)	−1 (−2, 0)	−1.211	0.226
Hospital stay, median (Q1, Q3), d	11 (10–12)	10 (9–13)	−1 (−2, 0)	−1.324	0.185

Group G, gelatin sponge sustained release group; group A, artificial dura mater sustained release group; Median difference = Group A− Group G. Negative values favor Group A. RR, Group A/Group G. RR > 1 favors Group A. RR, relative risk; CI, confidence interval; PONV, postoperative nausea and vomiting; PSQI, Pittsburgh sleep quality index. Compared with group G.

* *P* < 0.05.

**TABLE 4:** Comparison of hemodynamics between the two groups.

Outcomes	T0	T1	T2	T3
MAP of patients at various time points, mean (SD), mmHg				
Group G (n = 35)	92.03 (8.32)	85.86 (9.64)	89.63 (8.00)	83.37 (6.96)
Group A (n = 36)	91.28 (7.68)	84.78 (8.48)	87.89 (5.91)	82.67 (7.26)
*F*	0.156	0.251	1.091	0.174
*P*	0.694	0.618	0.300	0.678
*P-*group	*F =* 0.407	*P* = 0.525		
*P-*time	*F =* 65.211	*P* < 0.001		
*P-*group × time	*F =* 0.258	*P* = 0.771		
HR of patients at various time points, mean (SD), bpm				
Group G (n = 35)	77.74 (5.06)	75.06 (9.03)	76.89 (7.87)	80.74 (5.06)
Group A (n = 36)	77.03 (5.75)	73.42 (9.38)	75.08 (7.99)	79.03 (5.75)
*F*	0.308	0.563	0.917	1.775
*P*	0.580	0.455	0.342	0.187
*P-*group	*F =* 1.148	*P* = 0.288		
*P-*time	*F =* 16.574	*P* < 0.001		
*P-*group × time	*F =* 0.186	*P* = 0.808		

Group G, gelatin sponge group; group A, artificial dura mater group; SD, standard deviation; MAP, mean arterial pressure; HR, heart rate; mmHg, Millimeter of Mercury; BPM, Beat per minute. Compared with group G.

#### 3.3.4 Comparison of quality of recovery score: QoR-15 scale

**FIGURE 4:**
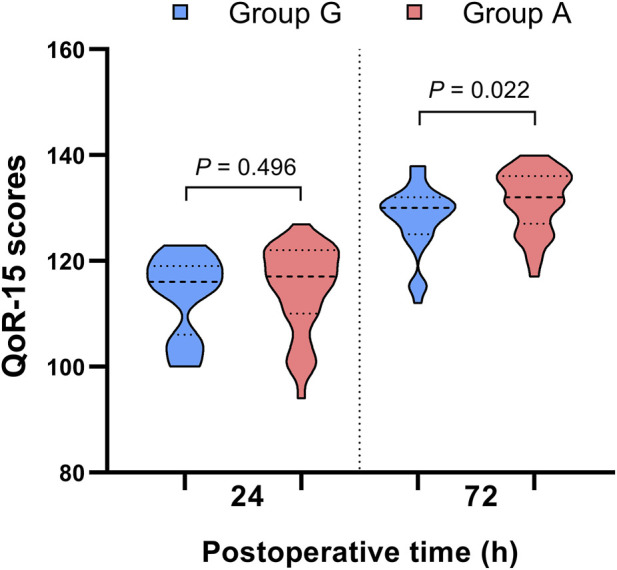
Group G, gelatin sponge group; group A, artificial dura mater group; QoR-15, Quality of Recovery-15; Comparison of QoR-15 scores between the two groups. Violin plots show the probability density of the data. The middle horizontal line represents the median, and the two outer lines represent Q1 and Q3.

The Mann–Whitney U test revealed that there was no significant difference in QoR-15 scores between group G and group A at 24 h after operation (*P* = 0.496). However, the QoR-15 scores of the two groups at 72 h after operation were 130 (125–132) and 132 (127–136), respectively, and the difference was statistically significant (*P* = 0.022). In the comparison of “have had a good sleep”, the score of group A was higher than that of group G (*P* = 0.038) (Table 5; Figure 4).

**TABLE 5:** Comparison of QoR-15 scores between the two groups.

QoR-15 scores	Patients, median (Q1, Q3)		*Z*	*P*
	Group G (n = 35)	Group A (n = 36)		
QoR-15 scores of all patients at 24 h				
Able to breathe easy	9 (8–9)	9 (8–9)	−0.312	0.755
Been able to enjoy food	7 (6–8)	7 (5.25–8.75)	−0.398	0.691
Feeling rested	8 (7–9)	7.5 (6.25–8.75)	−0.822	0.411
Have had a good sleep	7 (5–8)	7 (6–8)	−0.181	0.856
Able to look after personal toilet and hygiene unaided	3 (3–4)	3 (3–4)	−0.278	0.781
Able to communicate with family or friends	10 (9–10)	10 (9–10)	−0.376	0.707
Getting support from hospital doctors and nurses	9 (8–10)	9 (8–10)	−0.438	0.662
Able to return to work or usual home activities	3 (2–4)	3 (2–4)	−0.261	0.794
Feeling comfortable and in control	9 (8–9)	9 (8–10)	−0.826	0.409
Having a feeling of general wellbeing	9 (9–10)	9 (9–10)	−0.318	0.751
Moderate pain	7 (6–8)	7 (6–8)	−0.470	0.639
Severe pain	9 (8–10)	9 (8–10)	−0.129	0.898
Nausea or vomiting	8 (6–9)	8 (7–9)	−0.649	0.516
Feeling worried or anxious	9 (8–9)	9 (8–9)	−0.825	0.409
Feeling sad or depressed	10 (10–10)	10 (10–10)	−0.970	0.332
Total	116 (106–119)	117 (110–122)	−0.680	0.496
QoR-15 scores of all patients at 72 h				
Able to breathe easy	10 (9–10)	10 (9–10)	−0.577	0.564
Been able to enjoy food	8 (8–9)	9 (8–9.75)	−1.145	0.252
Feeling rested	8 (8–9)	9 (8–9)	−0.793	0.428
Have had a good sleep	7 (6–9)	8 (7.25–9)*	−2.074	0.038
Able to look after personal toilet and hygiene unaided	5 (5–6)	5 (5–6)	−0.487	0.626
Able to communicate with family or friends	10 (10–10)	10 (10–10)	−0.106	0.915
Getting support from hospital doctors and nurses	9 (8–10)	9 (9–10)	−0.817	0.414
Able to return to work or usual home activities	6 (6–7)	6.5 (6–7)	−1.223	0.221
Feeling comfortable and in control	9 (8–10)	9 (8–10)	−0.738	0.460
Having a feeling of general wellbeing	9 (9–10)	9.5 (9–10)	−0.520	0.603
Moderate pain	8 (7–9)	8.5 (8–10)	−1.656	0.098
Severe pain	9 (9–10)	10 (9–10)	−1.323	0.186
Nausea or vomiting	9 (9–10)	10 (9–10)	−0.510	0.610
Feeling worried or anxious	9 (9–10)	10 (9–10)	−1.800	0.072
Feeling sad or depressed	10 (10–10)	10 (10–10)	−0.787	0.431
Total	130 (125–132)	132 (127–136)^*^	−2.283	0.022

Group G, gelatin sponge group; group A, artificial dura mater group; QoR-15, Quality of Recovery-15; Compared with group G.

* *P* < 0.05.

## 4 Discussion

This study evaluated the efficacy of gelatin sponge and artificial dura mater as two distinct biomaterial carriers of hydromorphone following lumbar fusion surgery. Compared with gelatin sponge, artificial dura mater is already widely employed as a mature material for managing dural and cerebrospinal fluid leakage (Bai et al., 2013; Sun et al., 2024); furthermore, newer iterations of artificial dura mater have demonstrated positive therapeutic effects in repairing central nervous system injuries (Yang et al., 2025). Therefore, it may be both safe and clinically meaningful to utilize artificial dura mater for conventional dural repair while simultaneously leveraging its role as a hydromorphone carrier. The results of this study indicated that the perioperative hemodynamic stability and related safety profiles were comparable between the two groups. No significant difference was observed in the resting pain AUC from the PACU to 72 h postoperatively between the two groups (Median difference, −24; 95% CI, −36 to 0; *P* = 0.135). In contrast, a difference was noted in the movement-related pain AUC_PACU-72 h_ (Median difference, −24; 95% CI, −36 to 0; *P* = 0.045). The apparent discrepancy between the confidence interval boundary and the *P-*value is a recognized phenomenon of the Hodges-Lehmann estimator in the presence of tied observations. Sensitivity analyses confirmed the robustness of the primary results. Specifically, Group A demonstrated lower VAS score during movement at the 72-h postoperative mark (Median difference, −1; 95% CI, −1 to 0; *P* = 0.004), indicating that its control of movement-related pain may be better. Furthermore, patients in Group A reported lower PSQI scores (Median difference, −1; 95% CI, −1 to 0; *P* = 0.044) and higher total Quality of Recovery-15 (QoR-15) scores during the T3 period (*P* = 0.022), However, since the minimum clinically important differences for indicators such as AUC_PACU-72_ _h_, PSQI, or QoR-15 were not pre-specified under active conditions, the clinical relevance of these moderate differences necessitates further confirmation.

There was no significant difference in postoperative VAS scores at rest between the two groups (*P* > 0.05), and overall satisfaction was also comparable (*P* = 0.36). Additionally, no marked differences were observed in hemodynamic parameters or the incidence of most adverse reactions between the groups (*P* > 0.05). This indicates that both carriers may serve as drug containers for hydromorphone, providing a possible solution for pain relief at rest in PLIF patients. These findings provide some evidence for the clinical application of the two carriers, while indicating their interchangeability in terms of resting analgesia efficacy and safety. Although the resting pain relief effect is comparable, the artificial dura mater group may have certain advantages in functional recovery related indicators. The results of this study show that there is no significant difference in the early postoperative (24 h and 48 h) VAS scores during movement between the two groups. However, it is worth noting that the VAS scores during movement of the artificial dura mater group are lower at 72 h after surgery. Exercise induced pain - a key indicator for evaluating whether patients can engage in effective functional exercise early after surgery - has been widely included in the postoperative pain assessment system by multiple studies (Zhang et al., 2021; Tavanaei et al., 2022), and this time difference may have certain clinical significance. At the time point of 72 h after surgery, the drug release of the gelatin sponge group may have entered a declining stage, while the artificial dura mater group may still have a certain concentration of local drugs, which allows it to maintain a certain advantage during movements such as turning over and coughing. This phenomenon may be related to the physical structural characteristics of the artificial dura mater, which are denser and have a slower degradation rate. Similarly, postoperative sleep quality is closely associated with overall recovery (Hirai et al., 2025), and pain is a significant factor disrupting postoperative sleep (Li et al., 2025). This study observed that the artificial dura mater group exhibited better scores on both the Pittsburgh Sleep Quality Index (PSQI) and the single-item score for ‘had a good sleep’ from the QoR-15 scale at 72 h postoperatively (Campfort et al., 2022). This improvement may be the result of enhanced pain control. Quality sleep not only serves as a core component of patient comfort but also establishes the physiological foundation for tissue repair (Nassar et al., 2025), immune function maintenance (Lanza et al., 2024), stress response alleviation (Fukuoka et al., 2022), and emotional state improvement (Sin et al., 2021).

The relationship between improved sleep quality and overall recovery during the postoperative rehabilitation process is well-established. Studies indicate that the preoperative administration of opipramol can significantly enhance patients’ subjective sleep quality, potentially exerting a beneficial effect on postoperative recovery (Hueppe et al., 2011). Similarly, low-dose clonidine has been shown to improve sleep quality following surgery, further emphasizing the critical role of sleep quality in postoperative rehabilitation (Liu et al., 2024). Furthermore, research has demonstrated that the use of esketamine can enhance patients’ QoR-15 scores on the first and third postoperative days, while simultaneously improving sleep quality and reducing pain scores (Zhu et al., 2022). These findings align with those observed in the artificial dura mater group, highlighting the necessity of enhancing sleep quality to facilitate overall recovery. Additionally, a significant correlation has been identified between poor postoperative sleep quality and impaired wound healing, particularly from the third postoperative day onward. This observation corresponds with the artificial dura mater group’s elevated total QoR-15 scores on the third postoperative day (Das et al., 2025). Moreover, the variation in positive sleep-related outcomes primarily drove this difference, further confirming the existence of a positive feedback loop characterized by “pain control—sleep improvement—overall recovery” (Crooks et al., 2025). Therefore, enhancing pain management quality not only improves postoperative sleep but also promotes overall recovery. These findings provide a basis for clinical practice, suggesting that prioritizing improved sleep quality as an important objective during postoperative rehabilitation may lead to better recovery outcomes.

The clinical significance of this study lies in its expansion of postoperative analgesia strategies from merely assessing whether analgesia is adequate to understanding how analgesia facilitates functional recovery. Within the framework of enhanced recovery after surgery (ERAS) in spinal surgery (Corniola et al., 2020), enabling patients to engage in early and pain-free functional exercises is a core objective. Our results suggest that the choice of carrier may serve as a refined tool for analgesia modulation. The artificial dura mater, characterized by its physiologically compatible barrier properties, controllable degradation rate, and excellent tissue compatibility, may provide a more favorable carrier-based local delivery microenvironment for hydromorphone. Thus, artificial dura mater carriers may serve as a tool for postoperative analgesia regulation in PLIF patients, providing a new option for multimodal analgesia after spinal surgery (Yu et al., 2025).

## 5 Limitations

This study presents several limitations that warrant consideration. First, as a single-center study, the sample size may restrict our ability to detect more subtle differences in secondary outcomes. Secondly, although artificial dura mater is associated with better clinical outcomes, this study did not directly measure the drug concentration-time curve within the surgical area, nor did it conduct *in vitro* release kinetics testing on the artificial dura mater. Consequently, the discussion surrounding the ‘sustained-release mechanism’ remains speculative, based solely on clinical outcomes, and definitive pharmacokinetic conclusions cannot be drawn. Future research could employ *in vitro* release experiments to validate the drug release characteristics of different carriers. Third, although the gelatin sponge and artificial dura mater had identical surface areas in this experiment, the comparison between different carriers does not constitute a ‘pure carrier comparison’ since factors such as contact tightness with the dura and placement position are not fully equivalent to those of the artificial dura mater. Thus, the observed differences in effects may partially arise from variations in physical properties. Fourth, all patients had epidural drainage tubes postoperatively, and this drainage may influence the actual quantity and duration of the drug reaching target tissues through mechanisms such as dilution, washing, or removal of locally released drugs. This study did not monitor drug concentrations in drainage fluid or analyze the correlation between drainage volume and clinical outcomes, making it impossible to ascertain whether the observed efficacy originated from the carrier’s release properties or residual exposure affected by drainage. Fifth, although the primary outcome findings were robust in an ITT sensitivity analysis using multiple imputation, the potential impact of missing data on secondary outcomes remains unassessed. Sixth, the PSQI is typically utilized to assess patients’ sleep quality over a one-month period. However, this study, referencing relevant literature (Wang et al., 2024; Shi et al., 2025), applies the PSQI specifically to evaluate acute postoperative sleep during the initial 3 days. Finally, postoperative analgesia is influenced by multiple factors, and despite standardized management protocols, individual differences in pain sensitivity and motivation to recover may have influenced the results. Furthermore, the follow-up period was limited to 72 h, and the long-term analgesic effects remain to be evaluated. Meanwhile, outcomes such as neurological deficits, urinary retention, wound infection, cerebrospinal fluid leakage, epidural hematoma, duration of drainage and removal, or reoperation were not collected, all of which could potentially influence the final conclusions.

## 6 Conclusion

In this single-center randomized trial, both artificial dura mater and gelatin sponge can serve as carrier options for local hydromorphone delivery following PLIF. Compared with the gelatin sponge group, patients in the artificial dura mater group not only exhibited a lower AUC of the time-VAS curve during movement from PACU to 72 h postoperatively, but also achieved a higher early postoperative QoR-15 score, with no increase in adverse events. However, the clinical relevance of this reduction requires further investigation. These findings require validation in multicenter studies involving more diverse populations to establish broader generalizability.

## Data Availability

The original contributions presented in the study are included in the article/Supplementary Material, further inquiries can be directed to the corresponding authors.

## Glossary

ASA: American Society of Anaesthesiologists
AUC: Area under the curve
BMI: Body Mass Index
CRP: C-reactive protein
ERAS: Enhanced recovery after surgery
HR: Heart rate
MAP: Mean arterial pressure
mITT: Modified intention-to-treat
PLT: Platelet
PONV: Postoperative nausea and vomiting
PSQI: Pittsburgh Sleep Quality Index
PLIF: Posterior lumbar interbody fusion
PCEA: Patient-controlled epidural analgesia
PCA: Patient-controlled analgesia
PACU: Post Anesthesia Care Unit
QoR-15: Quality of Recovery-15
Hb: Hemoglobin
WBC: White blood cells
VAS: Visual Analogue Scale
RestVAS: Visual analog scale at rest
MoveVAS: Visual analog scale during movement
M: Median
MI: Multiple imputation
MD: mean difference
NSAIDs: Nonsteroidal anti-inflammatory drugs
IQR: Interquartile range
IV-PCA: intravenous patient-controlled analgesia
ITT: Intention-to-treat
SD: Standard deviation
SpO2: Pulse oximetry
SPSS: 26.0Statistical Product and Service Solutions 26.0
