# Supplementary material for: Exploring the analgesic effect of artificial dura mater as a carrier for local hydromorphone delivery in posterior lumbar interbody fusion: a randomized controlled trial
Source: Front Pharmacol. 2026 May 25;17:1816434. doi: 10.3389/fphar.2026.1816434 (PMC13243240; doi:10.3389/fphar.2026.1816434)
Supplement: Supplementary file 1 [file Table1.docx]

Supplementary Material

# Supplementary Tables

Table S1. Demographic and perioperative characteristics of the All-Randomized Population.

| Characteristic | Patients, mean (SD) | | *Z/t/χ2* | *P* |
| --- | --- | --- | --- | --- |
|  | Group G (n=37) | Group A (n=37) |  |  |
| Age, y | 57.54 (8.45) | 54.59 (10.73) | 1.312 | 0.194 |
| Height, median (Q1, Q3), cm | 160 (151.5-167.5) | 162 (155-168) | -0.417 | 0.676 |
| Weight, median (Q1, Q3), kg | 60 (55-66.5） | 60 (54.5-68） | -0.054 | 0.957 |
| WBC, 10^9/L | 6.67 (1.86) | 7.31 (2.53) | -1.238 | 0.220 |
| HGB, g/L | 131.73 (15.59) | 134.76 (15.96) | -0.825 | 0.412 |
| PLT, 10^9/L | 237.14 (72.73) | 254.65 (74.58) | -1.023 | 0.310 |
| Albumin, g/L | 40.42 (3.08) | 41.32 (3.55) | -1.162 | 0.249 |
| CRP, median (Q1, Q3), mg/L | 1.75 (1.41-2.95) | 1.86 (1.37-4.09) | 0.151 | 0.880 |
| BMI, kg/m² | 23.81 (3.04) | 23.33 (2.62) | 0.728 | 0.469 |
| Infusion volume, median (Q1, Q3), ml | 2000 (1500-2000) | 1800 (1500-2000) | -0.455 | 0.649 |
| Urine volume, median (Q1, Q3), ml | 300 (275-500) | 300 (200-400) | -1.618 | 0.106 |
| Blood loss, median (Q1, Q3), ml | 100 (100-200) | 100 (100-200) | -0.941 | 0.347 |
| SPO_2_, median (Q1, Q3), % | 98 (97-99) | 98 (98-99) | -0.051 | 0.959 |
| Surgical duration, median (Q1, Q3), min | 164 (137-190) | 150 (137-184) | -0.919 | 0.358 |
| Preoperative PSQI score, median (Q1, Q3) | 8 (6-9) | 7.5 (6-9) | -0.384 | 0.701 |
| Preoperative QoR-15 score, median (Q1, Q3) | 145 (143-147) | 144 (143-147) | -0.759 | 0.448 |
| Preoperative RestVAS score, median (Q1, Q3) | 1 (1-2) | 1 (1-3) | -0.193 | 0.847 |
| Diabetes, No. (%) | 3 (8.1) | 2 (5.4) | < 0.01 | > 0.99 |
| Hypertension, No. (%) | 7 (18.9) | 11 (29.7) | 1.175 | 0.278 |
| Gender | | | | |
| Male, No. (%) | 17 (45.9) | 19 (51.4) | 0.216 | 0.642 |
| Female, No. (%) | 20 (54.1) | 18 (48.6) |  |  |

Baseline characteristics of the modified intention-to-treat population were consistent with the all-randomized population. Group G: gelatin sponge group, group A: artificial dura mater group. PLT, Platelet; HGB, Hemoglobin; WBC, White blood cells; SPO2, Pulse Oxygen Saturation; BMI, Body mass index; CRP, C-reactive protein; SD, Standard deviation; RestVAS, visual analog scale at rest; PSQI, Pittsburgh sleep quality index; QoR-15, **Quality of Recovery-15**. Compared with group G, ^*^*P* < 0.05.
